# Coping strategies in young people during the COVID-19 pandemic: rapid review

**DOI:** 10.1192/bjb.2024.49

**Published:** 2025-08

**Authors:** Ranjita Howard, Harshini Manohar, Shekhar Seshadri, Aditya Sharma

**Affiliations:** 1 NHS England Education North East, Newcastle upon Tyne, UK; 2 National Institute of Mental Health and Neurosciences (NIMHANS), Bangalore, India; 3 Translational and Clinical Research Institute, Newcastle University, UK

**Keywords:** COVID-19, coping strategies, psychological challenges, child and adolescent mental health services

## Abstract

**Aims and method:**

To better understand factors supporting young people's (age <18 years) mental health during pandemic-type conditions, we aimed to identify whether coping strategies adopted during the COVID-19 pandemic could be dichotomised according to manifesting positive or negative psychological outcomes. Medline, EMBASE, CINAHL, PsycINFO, Scopus and ASSIA databases were used to identify empirical studies that examined coping strategies used by young people experiencing psychological challenges during COVID-19.

**Results:**

Twenty-five international studies were included, identifying that coping strategies adopted could be significantly dichotomised according to reducing or exacerbating psychological challenges. Positive coping strategies were proactive and solutions-oriented, whereas negative coping strategies were more avoidant and emotion-oriented.

**Clinical implications:**

An internal locus of control may account for why adolescents exercised more proactive coping compared with their younger counterparts, although parents of younger children may offset the impact of stressors by drawing on a proposed coping framework emphasising proactivity and engagement. This would be an invaluable addition to future pandemic preparedness planning cycles.

Although less physically affected by the COVID-19 pandemic than adults, the rising levels of anxiety,^1–7^ depression,^2,6–8^ stress,^9–11^ suicidal ideation,^12,13^ attention-deficit hyperactivity disorder^14,15^ and autism^16–18^ during the peak of the pandemic suggest that the mental health of young people (aged <18 years) was more affected.^19,20^ Given this impact, it is imperative to understand those factors that may help young people better manage through pandemic-like conditions and beyond, and one of the burgeoning areas of research is how young people cope with the stressors they face. Coping behaviour has been characterised by one's capacity to either engage a stressor or avoid it completely via the adoption of specific coping strategies.^21^ Originating from Lazarus and Folkman,^22^ such coping strategies are generally dichotomised as yielding positive outcomes, through exercising solutions-oriented, help-seeking or adaptive cognitive resources; or negative outcomes, through exercising avoidant-oriented, emotion-focused or maladaptive cognitive resources.^22–26^ More specifically, coping strategies that engage a stressor involve either proactive practices (primary control coping), such as listening to and taking advice from experts, or cognitive practices (secondary control coping) that allow one to adapt their response to the stressor, such as positively appraising or reframing its impact.^27,28^ Conversely, strategies that avoid or disengage one from a stressor involve efforts to orientate away from such, including denying a stressor's existence, suppressing one's emotions, withdrawal from others and substance misuse.^24,27^

In relation to child and adolescent research, both primary and secondary control coping have been significantly associated with the reduction of a range of psychological challenges in young people, including stress, anxiety, depression and loneliness,^29–31^ compared with avoidant strategies, which tend to exacerbate such.^30,31,63,64^ Moreover, this dichotomy may also extend to differences among children and adolescent populations who may manifest their coping behaviours differently, given that adolescents utilise more complex cognitive processes (i.e. internal locus of control) compared with younger children, who tend to cope more incidentally and are more reliant upon external sources (i.e. parental reactivity).^32–35^

## Method

### Aim

The aim of this review was to identify whether coping strategies employed by young people during the pandemic could be positively or negatively dichotomised in terms of significantly reducing or exacerbating psychological challenges, and whether there was a difference between children and adolescents in terms of the adoption of specific coping strategies. The development of a coping framework to offset the impact of tumultuous stressors as a consequence would no doubt be an invaluable addition to any future pandemic preparedness planning cycle.

### Search methodology

Medline, EMBASE, CINAHL, PsycINFO, Scopus and ASSIA databases were searched in March 2021. With respect to the coping strategies adopted, we used the following keywords: ‘coping’, ‘support’, ‘avoidance’, ‘help-seeking’, ‘problem-solving’, ‘stress management’, ‘distraction’, ‘escapism’, ‘resilience’, ‘adjustment’, ‘adaptive’ and ‘cognitive restructuring’ (Supplementary File 1 available at https://doi.org/10.1192/bjb.2024.49). Papers were included if they were empirical, peer-reviewed, available in English, published during the pandemic, included participants aged <18 years who were experiencing psychological challenges, and recorded data relevant to any coping strategies adopted.

### Screening and quality assessment

Following electronic and manual searches and consistent with Cochrane guidelines for rapid reviews, articles were independently screened by the first (R.H.) and second author (H.M.), and filtrated to potentially relevant papers, which were fully reviewed by R.H. and H.M. according to the inclusion criteria. Study quality, completed by R.H. and cross-checked by H.M., were assessed according to Strengthening the Reporting of Observational Studies in Epidemiology (STROBE) reporting guidelines (Supplementary File 2 available at https://doi.org/10.1192/bjb.2024.49). Any discrepancies or non-consensus during the screening and quality assessment process were resolved collaboratively and with the entire research team when necessary.

### Search outcome

**Fig. 1:**
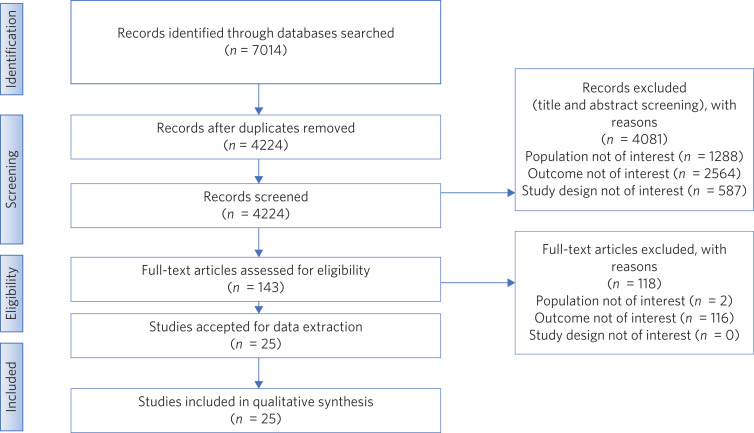
Flow diagram illustrating search strategy for the review.

A total of 7014 studies were found from the original electronic search, of which 25 met the inclusion criteria and were included in this review (see Fig. 1).

## Results

### Study design

Of the included studies, 22 were cross-sectional,^36–57^ one was longitudinal,^58^ one was mixed^59^ and one was interventional.^60^

### Countries of origin

Studies originated from China,^41,47,55,57,60^ the USA,^39,45,48,49,54^ Spain,^40,51^ Italy,^46,53^ Canada,^43^ India,^42^ Turkey,^38^ the UK,^59^ Belgium,^37^ Philippines,^36^ Russia,^44^ Holland^58^ and Qatar.^56^ Two studies involved a combination of countries: the USA and Puerto Rico;^50^ and Spain, Italy and Portugal.^52^

### Populations

The total number of participants from included studies was 25 157 (aged 0–18 years). Five studies sampled children exclusively (aged <13 years),^36,40,46,55,58^ 11 sampled adolescents exclusively (aged 13–18 years)^37,39,42–44,47,49,50,53,57,59^ and nine sampled both children and adolescents.^38,41,45,48,51,52,54,56,60^ Regarding psychological challenges, 15 studies assessed symptoms of anxiety,^37–39,41,44,46,47,51–53,55–57,59,60^ 14 assessed symptoms of decreased mood^37,41,43–47,49,51,52,55–57,60^ and eight assessed symptoms of stress^36,39,42,49,50,54,57,59^. Internalisation and externalisation of emotions, adjustment, cognitive and behavioural alterations, irritability and self-harm were also assessed.

### Study aims

The aim of included studies were to explore the efficacious employment of coping strategies generally,^36,40–42,45,47,52,53,56,57,59^ and regarding specific coping strategies, including communicating with family,^38,43,44,54^ communicating with friends,^38,43,44^ utilising social media or internet use,^37,43,44^ engaging in schoolwork/online learning,^43,47,48,55^ self-care practices,^38,50^ pet relations,^3^ physical activity^50^ and mindfulness.^60^ Additional areas included the influence of parental reactivity;^40,55,58^ demographic characteristics, including age^40^ and geography;^46,52^ coping and resilience training;^51^ and pre-existing challenges, such as neurodevelopment difficulties^39,48^ and physical health difficulties.^38^

### Measures

Measures predominantly assessed coping style/strategy, and symptoms relating to anxiety, depression, emotional regulation and life satisfaction. Two studies measured coping style/strategy using the Brief Coping Orientation to Problems Experienced (COPE) Scale,^37,59^ two used the KidCOPE inventory,^40,48^ two used a scale based on Parker and Endler's (1992) theorem^46,52^ and others utilised the Children's Coping Strategies Checklist,^40^ the Coping Style Scale,^41^ the Coping Strategies Inventory,^45^ the Trait Coping Style Questionnaire,^60^ the Coping Inventory to COVID-19 and Home Confinement in Children and Adolescents,^51^ the Coping with Children's Negative Emotions Scale (CCNES),^55^ the Coping Style Questionnaire (CSQ)^57^ and a scale influenced by Edge and Sherwood.^56^ The remaining studies assessed coping style/strategy with bespoke instrumentation.

Regarding anxiety symptoms, the State-Trait Anxiety Inventory for Children (STAI-C),^39^ the Spence Child Anxiety Scale (SCAS),^41,56^ the Swine Flu Anxiety Scale^43^ and the Generalised Anxiety Disorder-7 (GAD-7)^47^ scales were used, and decreased mood symptoms were assessed with several pre-existing scales, including the Child Depression Inventory,^41^ the UCLA Loneliness Scale,^43^ the Kutcher Adolescent Depression Scale (KADS)^56^ and the Patient Health Questionnaire-9 (PHQ-9).^47^ Stress symptoms were assessed by the Responses to Stress Questionnaire (RSQ)^39^ and the Perceived Stress Scale (PSS).^58^

Pre-existing measures were also used to assess symptoms relating to emotional regulation, resilience and poor well-being. These included the Cognitive Emotion Regulation Questionnaire (CERQ)^58^ and Difficulties in Emotion Regulation Scale-COVID-19^48^; the Connor–Davidson Resilience Scale^40^ and Brief Resilience Scale^57^; and the Satisfaction with Life Scale (SWLS)^44^ and Well-Being Index (WHO-5),^44^ respectively.

The utilisation of a range of bespoke instrumentation to evaluate symptoms of anxiety,^52,53^ low mood,^48,52^ stress^36,49^ and loneliness^49^ were also utilised.

### Synthesis of results

#### Positive coping strategies

Of the 25 studies reviewed, 17 identified positive coping strategies among children and adolescents that were significantly responsible for the reduction of a psychological challenge (see Table 1). The majority of coping strategies adopted were proactive, problem-oriented and engaging with respect to the pandemic stressor, and associated with reduced symptoms of anxiety, depression, loneliness, stress, sleep problems, and behavioural and cognitive alterations, at the noted significance levels.

**Table 1:** Positive psychological changes when a coping strategy is adopted by children and adolescents post COVID-19 onset

Author	Cohort age, years	Sample size	Positive coping strategy	Psychological challenge reduced at significant level post COVID-19 onset	Primary or secondary control coping
Cauberghe et al^37^	13–18	2165	Active and Adaptive coping (social media for positive appraisal and cognitive restructuring; social media to connect with peers and family, and for humour)	Anxiety, loneliness*	Primary and secondary
Cenk et al^38^	All	267	Active and Adaptive coping (engagement in activities, finding new hobbies, communicating with friends via social media)	Anxiety***	Primary and secondary
Corbett et al^39^	13–18	122	Adaptive coping skills (engagement; positive appraisal and cognitive restructuring – acceptance, reframing)	Anxiety and stress*	Secondary
Domínguez-Álvarez et al^40^	<13	1123	Active coping skills (problem-focused coping)	Psychosocial adjustment*	Primary
Duan et al^41^	All	3613	Active coping skills (problem-focused coping)	Depression**	Primary
Ellis et al^43^	13–18	1054	Adaptive coping (communication with loved ones, virtual connection with friends, physical exercise)	Loneliness*	Secondary
Gerasimova and Kholmogorova^44^	13–18	88	Adaptive coping (communication with loved ones, help-seeking, less internet usage)	Depression, loneliness, anxiety****	Secondary
Hussong et al^45^	All	88	Active and adaptive coping (problem-solving; positive appraisal and cognitive restructuring – self efficacy)	Lesser increase in symptoms***	Primary and secondary
Li et al^47^	13–18	850	Active coping skills (problem-based coping – taking treatment/vaccines)	Online learning satisfaction, anxiety, depression****	Primary
Liang et al^46^	<13	1074	Active and adaptive coping (problem-solving; positive appraisal and cognitive restructuring – acceptance, advantages of being at home)	Anxiety, * mood***	Primary and secondary
Liu et al^60^	All	121	Adaptive coping skills (mindfulness – perceives world more objectively; improves metacognitive abilities; meaning of life)	Anxiety, depression, internet addiction****	Secondary
McFayden et al^48^	All	49	Adaptive coping skills (i.e. engagement in more schoolwork)	Lesser increase in symptoms***	Secondary
Orgilés et al (20)^51^	All	96	Active and adaptive coping (problem-solving; cognitive restructuring and appraisal)	Anxiety, mood, sleep problems, cognitive alterations*	Primary and secondary
Orgilés et al (21)^52^	All	1480	Adaptive coping (positive appraisal and cognitive restructuring; acceptance)	Symptoms in general, in particular mood, sleep problems, behavioural, and cognitive alterations***	Secondary
Pigaiani et al^53^	13–18	306	Active and adaptive coping (routine/structured activities; help-seeking; social support)	Better well-being****	Primary and secondary
Zainel et al^56^	All	6608	Active and adaptive coping (adherence with regulations; information retrieval from legitimate sources; spirituality; family time)	Depression****	Primary and secondary
Zhang et al^57^	13–18	1025	Active and adaptive coping (positive appraisal and cognitive restructuring; problem-solving; help-seeking)	Depression, anxiety and stress*	Primary and secondary

* *P* = 0.001, **P = 0.005, ***P = 0.01, ****P = 0.05.

##### Solutions-oriented coping strategies

Complying with regulations, taking appropriate medication and vaccines, help-seeking and drawing on legitimate resources were solutions-oriented strategies that were found to be significant. For example, Zainel et al^56^ found that the majority adhered to governmental regulations during quarantine and sought out accurate information from official channels, strategies that were significantly associated with the reduction of depressive symptoms. Cenk et al,^38^ in their comparison of 132 youths with cystic fibrosis with 135 healthy equivalents, found that through following infection control guidelines such as wearing masks and washing hands, the former presented with lower anxiety symptoms than their healthy peers. Although not reporting on a significant association, Tambling et al,^54^ in their qualitative analysis of parent-reported interactions with their children, demonstrated the positive role of parenting with respect to parents being sources of coping socialisation through making personal hygiene fun and engaging for their children.

##### Positive appraisal and cognitive restructuring

Acceptance of the situation, reframing the problem, seeing the advantages of being at home and using humour online were also found to be significantly efficacious. Liang et al,^46^ for example, found that acceptance of the situation (62%), seeking affection (36%) and positively appraising the benefits of being at home (36%) were responsible for the reduction of anxiety and mood symptoms for those in the least affected areas. Similarly, Corbett et al,^39^ who compared typically developed youths and those with autism spectrum disorder, found that typically developed youths adopted more acceptance, reframing and positive thinking strategies than those with autism spectrum disorder, resulting in significant reductions of stress and anxiety.

##### Communicating with family and friends

Drawing on support and advice from friends via social media, and spending more time with loved ones were also positively significant. Ellis et al,^43^ for example, found that spending time with family, whether face to face or via video messaging, and virtually connecting with friends, was significantly associated with a reduction in loneliness and depression. Additionally, Gerasimova and Kholmogorova^44^ found that regular interaction with family was significantly associated with less loneliness and better psychological well-being, and Pigaiani et al^53^ found that better well-being was associated with receiving support from family, allowing individuals to share their feelings and re-evaluate family relationships.

##### Engaging in structured activities

Activities such as schoolwork, taking on a new hobby, exercising more and engaging in mindful or spiritual activities were also significantly associated with reduced psychological challenges across our sample. Pigaiani et al,^53^ for example, found that engaging in structured activities (schoolwork) and developing new interests (physical activity) was significantly associated with better well-being. Liu et al,^60^ who reported on the effects of a logotherapy-based mindfulness intervention on internet addiction, found that, as well as reducing internet addiction, the mindfulness intervention also significantly alleviated anxiety and depression levels compared with the those in the control group. Regular engagement in spiritual activities (Zainel et al^56^) exercise and the establishment of a routine (O'Brien et al^50^) were also related to positive well-being, although O'Brien et al's findings were based on thematic analysis and thus not grounded in significantly statistical data with respect to any psychological outcome.

#### Negative coping strategies

Of the 25 studies reviewed, 14 identified negative coping strategies among children and adolescents that were significantly responsible for the increase of a psychological challenge (see Table 2). The majority of coping strategies adopted were emotion-oriented, self-critical and avoidant with respect to the pandemic stressor, and associated with the exacerbation of anxiety, depression, mood disturbances, stress, internalisation and externalisation of emotions, and behavioural and cognitive alterations, at the noted significance levels.

**Table 2:** Negative psychological changes when a coping strategy is adopted by children and adolescents post COVID-19 onset

Author	Cohort age, years	Sample size	Negative coping strategy	Psychological challenge increased at significant level post COVID-19 onset	Emotional/avoidant coping
Achterberg et al^58^	<13	151	Perceived stress; rumination; parental emotion- oriented reactions (over-reactivity)	Stress*	Emotional and avoidant
Dewa et al^59^	13–18	360	Disengagement; self-blame, substance misuse	Anxiety,* stress*	Emotional
Domínguez-Álvarez et al^40^	<13	1123	Disengagement; parental emotion-oriented reactions (fear of future)	Internalising* and externalising problems*	Emotional
Duan et al^41^	All	3613	Excessive smartphone usage; internet usage	Anxiety,* mood disturbances*	Avoidant
Ellis et al^43^	13–18	1054	Excessive social media/virtual time with friends (co-rumination); little time with family; less time with schoolwork; little physical activity	Mood disturbances*	Emotional and avoidant
Hussong et al^45^	All	88	Negative self-appraisal – self-criticism; withdrawal	Internalising* and externalising problems*	Emotional
Li et al^47^	13–18	850	Emotion-oriented coping	Anxiety,*** mood disturbances***	Emotional
Liang et al^46^	<13	1074	Often talks about feelings, angry, seeks affection; avoidance-oriented coping (e.g. changes topic, acts nothing happening, disengagement)	Anxiety,* mood disturbances,** cognitive alterations****	Emotional and avoidant
McFayden et al^48^	All	49	Parental emotion-oriented reactions (psychopathology)	Cognitive alterations (disengagement/ concentration in schoolwork, remote learning)*	Emotional
Mueller et al^49^	All	357	Avoidance (dog ownership)	Loneliness (*P* = 0.008)	Avoidant
Orgilés et al (20)^51^	All	96	Negative self-appraisal	Anxiety,* mood disturbances,* sleep,*** cognitive alterations***	Emotional
Orgilés et al (21)^52^	All	1480	Avoidant –disengagement coping; negative self-appraisal)	Anxiety, mood disturbances, sleep, behavioural and cognitive alterations***	Emotional and avoidant
Wang et al^55^	<13	3280	Parental emotion-oriented reactions (punitive)	Depression,* loneliness***	Emotional
Zhang et al^57^	13–18	1025	Avoidance (keep feelings to self; avoiding situation; isolation)	Depression, anxiety, stress****	Avoidant

* *P* = 0.001, **P = 0.005, ***P = 0.01, ****P = 0.05.

##### Avoidance-oriented coping strategies

Denying the pandemic's existence, suppressing one's feelings, changing the topic of conversation and emotionally disengaging from events constituted avoidance-oriented strategies that were found to be significant. Zhang et al,^57^ for example, found that keeping feelings to oneself and avoiding the situation were significantly associated with depression, anxiety, stress and trauma-related stress. Moreover, Liang et al^46^ found that trying not to worry, denying the pandemic's existence and emotionally disengaging from the negative emotions exhibited by parents was associated with worsening levels of anxiety, mood and cognitive disturbances for those in the more affected areas. Employing avoidant responses to parental reactions to the pandemic (i.e. parental over-reactivity, parental fear of the future and punitive parenting) was common across several of our studies, responses that were again significantly associated with negative psychological implications.^40,55,58^

##### Negative appraisal and rumination

Expressing anger with the situation, blaming oneself, being self-critical and rumination were also found to be significantly efficacious. Hussong et al,^45^ for example, found that engaging in negative self-appraisal and self-criticism was significantly associated with a higher risk of internalisation and externalisation of emotions, and Dewa et al^59^ found that self-blame and a fastidious personality were significantly associated with anxiety and stress. Rumination was also found to be significantly associated with increased stress (Achterberg et al^58^), anxiety and depression (Orgilés et al^52^), and mood disturbances (Ellis et al^43^). Indeed, Ellis et al^43^ attributes the high levels of depression found among their adolescent sample to co-rumination or the excessive discussion of problems and concerns with friends on social media.

##### Social withdrawal

Withdrawal from loved ones, spending more virtual time with friends than face-to-face time with family and regarding pets as their primary social companion, were also negatively significant. Ellis et al,^43^ for example, found that although time on social media and other virtual connections had increased, 36% of adolescents spent less than 30 min a day face to face with family, which may account for the significantly high levels of depression among their sample. Moreover, Mueller et al^49^ found that despite spending more time with their pets to deal with loneliness, such adolescents experienced significantly higher levels of loneliness compared with pre-pandemic levels, possibly because it was at the expense of using more adaptive strategies such as spending time with family and friends.

##### Maladaptive activities

Substance misuse, excessive internet usage, excessive smartphone usage and spending less time on schoolwork and physical activity also significantly exacerbated respective psychological challenges across our sample. Ellis et al,^43^ for example, found low levels of physical activity among their adolescent sample (<60 min per day), which was significantly associated with high levels of loneliness. Duan et al^41^ found that smartphone and internet addiction (more than 5 h per day), evident within 30% of respondents, was associated with significant increases in depression. Substance misuse (Dewa et al^59^), spending less time on schoolwork (Ellis et al^43^), playing video games, sleeping and excessive television, alcohol and drug use (O'Brien et al^50^) were also found to be significantly maladaptive on young people's mental health, although O'Brien et al's findings were again not based on a statistically significant data-set with respect to any psychological outcome.

#### Differences between children and adolescents

A difference between children and adolescents in terms of the adoption of specific coping strategies was also indicated. Indeed, 64% of included studies that sampled adolescents exclusively were associated with the adoption of positive or more controlled coping strategies (i.e. solution-oriented coping,^47,57^ positive appraisal and restructuring,^37,39,57^ communication with family,^43,44^ structured activities^53^). This compares with 80% of included studies that sampled preadolescents and children exclusively and were associated with the adoption of negative or more avoidant coping strategies (i.e. parental reactivity,^40^ avoidance,^46^ rumination^58^).

## Discussion

The findings of this review suggest that the coping strategies adopted by young people during the peak of the pandemic could be significantly dichotomised according to either positive or negative psychological outcomes. Indeed, the adoption of solution-oriented coping strategies (following guidelines, information gathering),^37,38,40,41,45–47,51,53,54,56,57^ cognitive strategies (positive appraisal, reframing interpretation),^37,39,45,46,51,52,57^ supportive strategies (time with family, online peer support)^37,38,43,44,53,56,57^ and adaptive structure/distractions (schoolwork, exercise, spirituality, mindfulness)^38,43,48,53,56,60^ were significantly associated with a reduction of respective psychological challenges. Comparatively, the adoption of avoidant-oriented strategies (denying the pandemic, suppressing emotions, parental reactivity),^40,46,52,55,57,58^ negative appraisal strategies (blaming oneself, being excessively self-critical, rumination),^43,45,52,58,59^ social withdrawal^43,49^ and excessive indulgences (internet and smartphone usage, reduced exercise and schoolwork, substance misuse)^41,43,50,59^ were significantly associated with an exacerbation of respective psychological challenges.

Such findings are consistent with research showing that proactivity and engagement when dealing with stressors reduces a range of psychological challenges in young people, including stress, anxiety, depression and loneliness,^29,30,61,62^ whereas being avoidant and disengaging tends to exacerbate psychological challenges.^30,31,63,64^ Compas et al,^30^ for example, in their meta-analytic review of 212 studies (age range 5–19 years), found that both primary and secondary means of engaging a stressor significantly reduced internalising and externalising psychopathology. Conversely, Schäfer et al,^31^ in their meta-analytic review of 35 studies (age range 13–18 years), found that maladaptive coping strategies, such as avoidance, rumination, suppression and denial, significantly increased symptoms of psychopathology.

To explain such findings, it may be fruitful to draw on the control-based model of coping^65–67^ and the notion of locus of control,^33^ which proclaim that those able to maintain a sense of volition, self and coherence are more equipped at adapting to stressors that are tumultuous and emotionally disorienting.^68–71^ Indeed, being informed by a more constructive and consciously engaged cognitive process may underpin the efficacy of the positive coping strategies adopted by the youths sampled in this review,^69,70^ whereas more impulsive and insecure cognitive processes may underpin those adopting negative coping strategies.^72,73^ Such models may also explain why the majority of our studies that exclusively sampled adolescents were associated with the adoption of positive coping strategies, whereas the majority that exclusively sampled preadolescents and children were associated with the adoption of negative strategies. Indeed, in having a greater internal locus of control, it may be the case that adolescents have a greater sense of control over life events, resulting in the adoption of more proactive means of coping. This compares with younger children whose life events are influenced by factors externally, and thus are dependent on and reactive to the people around them, such as parents.^33^

In short, the findings of this review suggest that when dealing with stressors, particularly those that are as tumultuous as pandemic-like events, it is imperative that young people are encouraged to be as proactive and engaging as possible. Adherence to guidelines, help-seeking, spending time with family, socialising with friends, positively appraising events and engagement in healthy routines appear to represent a set of practices that should maintain a young person's well-being during such stressors (see Box 1). Given the difficulties for younger children to engage so constructively, it is critical that parents with younger children are able to provide them with cognitive, behavioural and emotional scaffolding through possibly drawing on the set of coping strategies mentioned. Indeed, interventions that can reduce parental psychopathology, that build parental resilience and compassionate expression, and that increase internal locus of control among parents themselves, may go a long way toward ensuring that younger children can also maintain a ‘sense of coherence’ when facing stressors that are incredibly tumultuous and disorienting.

Box 1 Coping best practice for young people during pandemic-type events

- Solutions-oriented
  - Adherence to guidelines
  - Help-seeking
  - Drawing on legitimate news sources
  - Healthy habits, e.g. exercise
- Positive appraisal/restructuring
  - Acceptance
  - Reframing the problem
  - Consider circumstantial advantages
  - Using humour
- Communicating with family and friends
  - Spending time with loved ones
  - Seeking online support from friends
- Engaging in structured activities
  - Schoolwork
  - Hobbies
  - Healthy habits, e.g. exercise
  - Spiritual/metacognitive activities

### Strengths and limitations

This review represents one of the few that explores the coping strategies adopted by young people during the peak of the COVID-19 pandemic. It presents evidence from a range of countries, provides data from a good number of studies, a range of age groups across childhood and adolescence, and conclusions are based on largely significant data-sets. Moreover, data collection and quality assessment adhered to the Cochrane and STROBE levels of scrutiny, respectively. Limitations include the restriction of studies accessible in English and the limited number of studies comparing an intervention with a control group, suggesting a lack of high-quality research in this area. Given that the data taken from the vast majority of studies were based on self-reported questionnaires, this also presents the problem of self-report bias and accuracy of recall. The heterogeneity of instruments used to assess coping strategy, as well as the conceptual frameworks that informed such, also made it difficult to compare across studies, thus jeopardising generalisable conclusions.

In conclusion, proactive and engaged coping appeared effective in reducing a range of psychological challenges among young people during the peak of the COVID-19 pandemic, whereas avoidant-oriented coping appeared to exacerbate such challenges. Advanced cognitive processes such as an internal locus of control may account for why adolescents tended to exercise coping strategies that are more proactive and constructive. Conversely, less secure cognitive processes based on an external locus of control may explain why younger children are drawn to more incidental coping means when facing extreme life stressors, although their sense of coherence could potentially be maintained by a degree of cognitive and behavioural scaffolding from their parents. Follow-up research that considers such variations and potential others (i.e. cultural, neuro-developmental vulnerabilities) would further elucidate coping differences across the child and adolescent mental health literature. However, this review also draws attention to the heterogenous nature of how coping as a body of research is defined and therefore measured, and this needs to be addressed to offset the methodological and conceptual stagnation the field of coping still finds itself in (see Compas et al^30^). Nevertheless, based on the findings of this review at least, a coping framework that is inherently proactive and engaging would serve as a protective factor towards the onset or exacerbation of psychological distress during pandemic-like episodes for children and adolescents, and this would serve as an invaluable addition to any future pandemic preparedness planning cycle.

## About the authors

**Ranjita Howard** is an ST6 Specialist Registrar with Child and Adolescent Mental Health Services, NHS England Education North East, Newcastle upon Tyne, UK. **Harshini Manohar** is an Assistant Professor in Child and Adolescent Mental Health Services, National Institute of Mental Health and Neurosciences (NIMHANS), Bangalore, India. **Shekhar Seshadri** is a Professor in Child and Adolescent Mental Health Services, National Institute of Mental Health and Neurosciences (NIMHANS), Bangalore, India. **Aditya Sharma** is a Clinical Senior Lecturer and Honorary Consultant Psychiatrist in Child and Adolescent Psychiatry at the Translational and Clinical Research Institute, Newcastle University, UK.

## Supporting information

Howard et al. supplementary material 1 Howard et al. supplementary material

Howard et al. supplementary material 2 Howard et al. supplementary material

## Data Availability

The data that support the findings of this study are available on request from the corresponding author, R.H.
