# Supplementary material for: Coping strategies in young people during the COVID-19 pandemic: rapid review
Source: BJPsych Bull. 2025 Aug;49(4):249–58. doi: 10.1192/bjb.2024.49 (PMC12314407; doi:10.1192/bjb.2024.49)
Supplement: Howard et al. supplementary material 1 — Howard et al. supplementary material [file S2056469424000494sup001.pdf]

Supplementary File 01: Database search terms used to look at the coping strategies amongst young people:

('young people' OR 'youth' OR 'adolescen*' OR 'young adult' OR 'teen*' OR 'child*' AND 'COVID') AND ('coping' OR 'support' OR 'avoidance' OR 'helpseeking' OR 'problem-solving' OR 'stress manag*' OR 'distraction' OR 'escap*' OR 'resilien*' OR 'adjust*' OR 'adapt*' OR 'cognitive restructuring')
