# Supplementary material for: Coping strategies in young people during the COVID-19 pandemic: rapid review
Source: BJPsych Bull. 2025 Aug;49(4):249–58. doi: 10.1192/bjb.2024.49 (PMC12314407; doi:10.1192/bjb.2024.49)
Supplement: Howard et al. supplementary material 2 — Howard et al. supplementary material [file S2056469424000494sup002.pdf]

Supplementary File 02: Study quality was assessed according to STROBE reporting guidelines

	Title and abstract	Background	Objectives	Study design	Setting	Participants (methods)	Variables	Data measurement	Bias	Study size	Quantitative variables	Statistical methods	Participants (results)	Descriptive data	Outcome data	Main results	Other analyses	Key results	Limitations	Interpretation	Generalisability	Funding
Achterberg et al	✓	✓	✓	✓	✓	✓	✓	✓	✓	✗	✓	✓	✓	✓	✓	✓	✗	✓	✓	✓	✓	✗
Agbing et al	✓	✓	✓	✓	✓	✓	✓	✓	✗	✗	✓	✓	✓	✓	✓	✓	✗	✓	✓	✓	✓	✓
Cauberghe et al	✓	✓	✓	✓	✓	✓	✓	✓	✗	✗	✓	✓	✓	✓	✓	✓	✗	✓	✓	✓	✓	✓
Cenk et al	✓	✓	✓	✓	✓	✓	✓	✓	✗	✗	✓	✓	✓	✓	✓	✓	✗	✓	✓	✓	✓	✗
Corbett et al	✓	✓	✓	✓	✓	✓	✓	✓	✗	✓	✓	✓	✓	✓	✓	✓	✗	✓	✓	✓	✓	✓
Dewa et al	✓	✓	✓	✓	✓	✓	✓	✓	✗	✗	✓	✓	✓	✓	✓	✓	✗	✓	✓	✓	✓	✓
Dominguez-Alvarez et al	✓	✓	✓	✓	✓	✓	✓	✓	✗	✗	✓	✓	✓	✓	✓	✓	✓	✓	✓	✓	✓	✓
Duan et al	✓	✓	✓	✓	✓	✓	✓	✓	✗	✗	✓	✓	✓	✓	✓	✓	✗	✓	✓	✓	✓	✓
Dumber et al	✓	✓	✓	✓	✓	✓	✓	✓	✗	✗	✓	✓	✓	✓	✓	✓	✗	✓	✗	✓	✗	✓
Ellis et al	✓	✓	✓	✓	✓	✓	✓	✓	✗	✗	✓	✓	✓	✓	✓	✓	✓	✓	✓	✓	✓	✓
Gerasimova et al	✓	✓	✓	✓	✓	✓	✓	✓	✗	✗	✓	✓	✓	✓	✓	✓	✗	✓	✓	✓	✓	✓
Hussong et al	✓	✓	✓	✓	✓	✓	✓	✓	✗	✗	✓	✓	✓	✓	✓	✓	✓	✓	✓	✓	✓	✓
Li et al	✓	✓	✓	✓	✓	✓	✓	✓	✗	✗	✓	✓	✓	✓	✓	✓	✗	✓	✓	✓	✓	✓
Liang et al	✓	✓	✓	✓	✓	✓	✓	✓	✗	✗	✓	✓	✓	✓	✓	✓	✓	✓	✓	✓	✓	✓
Liu et al	✓	✓	✓	✓	✓	✓	✓	✓	✗	✗	✓	✓	✓	✓	✓	✓	✗	✓	✗	✓	✓	✗
McFayden et al	✓	✓	✓	✓	✓	✓	✓	✓	✗	✗	✓	✓	✓	✓	✓	✓	✗	✓	✓	✓	✓	✗
Mueller et al	✓	✓	✓	✓	✓	✓	✓	✓	✗	✗	✓	✓	✓	✓	✓	✓	✗	✓	✓	✓	✓	✓
O'Brien et al	✓	✓	✓	✓	✓	✓	✓	✓	✗	✗	✓	✓	✓	✓	✓	✓	✗	✓	✓	✓	✓	✓
Orgiles and E et al	✓	✓	✓	✓	✓	✓	✓	✓	✗	✗	✓	✓	✓	✓	✓	✓	✗	✓	✓	✓	✓	✓
Orgiles et al	✓	✓	✓	✓	✓	✓	✓	✓	✗	✗	✓	✓	✓	✓	✓	✓	✓	✓	✓	✓	✓	✓
Pigaiani et al	✓	✓	✓	✓	✓	✓	✓	✓	✗	✗	✓	✓	✓	✓	✓	✓	✗	✓	✓	✓	✓	✓
Tambling et al	✓	✓	✓	✓	✓	✓	✓	✓	✗	✗	✓	✓	✓	✓	✓	✓	✗	✓	✓	✓	✓	✗
Wang et al	✓	✓	✓	✓	✓	✓	✓	✓	✗	✗	✓	✓	✓	✓	✓	✓	✗	✓	✓	✓	✓	✓
Zainel et al	✓	✓	✓	✓	✓	✓	✓	✓	✓	✓	✓	✓	✓	✓	✓	✓	✓	✓	✓	✓	✓	✗
Zhang et al	✓	✓	✓	✓	✓	✓	✓	✓	✗	✗	✓	✓	✓	✓	✓	✓	✓	✓	✓	✓	✓	✗
